# The effect of combined ultrasound stimulation and gastrodin on seizures in mice

**DOI:** 10.3389/fnins.2024.1499078

**Published:** 2024-11-22

**Authors:** Houminji Chen, Yuqing Miao, Haowen Duan, Shasha Yi, Zhengrong Lin, Yanwu Guo, Junjie Zou, Lili Niu

**Affiliations:** ^1^Guangdong Provincial Key Laboratory on Brain Function Repair and Regeneration, Department of Neurosurgery, The National Key Clinic Specialty, The Engineering Technology Research Center of Education Ministry of China, Zhujiang Hospital, Southern Medical University, Guangzhou, China; ^2^Institute of Biomedical and Health Engineering, Shenzhen Institute of Advanced Technology, Chinese Academy of Sciences, Shenzhen, China; ^3^The Key Laboratory of Biomedical Imaging Science and System, Chinese Academy of Sciences, Shenzhen, China

**Keywords:** focus ultrasound, neuromodulation, gastrodin, epilepsy, combination therapy

## Abstract

Both physiotherapy and medicine play essential roles in the treatment of epilepsy. The purpose of this research was to evaluate the efficacy of the combined therapy with focus ultrasound stimulation (FUS) and gastrodin (GTD) on seizures in a mouse model. Kainic acid-induced seizure mice were divided into five groups randomly: sham, FUS, saline + sham, GTD + sham and GTD + FUS. The results showed that combined therapy with ultrasound stimulation and gastrodin can significantly reduce the number and duration of seizures in GTD + FUS group. 9.4T magnetic resonance imaging and histologic staining results revealed the underlying mechanism of the combined therapy may be that ultrasound stimulation increases cell membrane permeability to increase GTD concentration in brain. In addition, we verified the safety of FUS combined with GTD therapy. This research provides a new strategy for neurological disorders combining treatment of physical neuromodulation and medicine.

## Introduction

70 million people suffer from epilepsy worldwide, making epilepsy one of the most prevalent neurological disorders (Thijs et al., 2019). Drugs are the major treatment for epilepsy; however, currently available antiepileptic drugs (AEDs) have limited efficacy (Chen et al., 2018; Perucca et al., 2023). Surgical resection of epileptic foci can be used in patients with refractory epilepsy whose epileptic focus can be localized. Neuromodulation techniques use physical means to stimulate the central or peripheral nervous system and are widely used as an essential strategy in epilepsy treatment (Haneef and Skrehot, 2023). In clinical practice, vagus nerve stimulation (VNS) has shown good improvement in seizures, while deep brain stimulation (DBS) can also be used to control abnormal discharges (Ryvlin et al., 2021; Thompson et al., 2021). However, these invasive neuromodulation modalities require surgical implantation of stimulation electrodes, and patients need to bear the risks and discomfort associated with surgery (Fisher, 2023).

Ultrasound, with complete non-invasiveness as its greatest advantage, has been used to neuromodulate Parkinson’s disease, Alzheimer’s disease and other neurological disorders (Zhang et al., 2019; Zhou et al., 2019; Wu et al., 2020). In epilepsy, ultrasound stimulation of focal brain slices in epileptic patients has been shown to inhibit seizure onset and propagation by modulating the excitatory/inhibitory output ratio (Lin et al., 2020). In the mouse and monkey models of epilepsy, ultrasound stimulation of epileptogenic regions located in the hippocampus or frontal lobe has been able to reduce seizure severity (Lin et al., 2019; Zou et al., 2020; Zou et al., 2021). Even extending to the peripheral nervous system, ultrasound modulation of the vagus nerve has exerted considerable anti-epileptic effects (Zou et al., 2024). In human motor cortex (M1), similar to findings in isolated brain slices, neuronal excitation/inhibition ratios can also be non-invasively modulated by ultrasound (Zhang et al., 2023). Overall, ultrasound neuromodulation is a promising modality for treatment of epilepsy. Neuromodulation techniques combined with pharmacotherapy have also shown potential application in the brain diseases. VNS, the most used neuromodulation therapy for epilepsy is reported to be effective when used in combination with AEDs to control drug-resistance epilepsy (DRE) patients’ seizure (Panebianco et al., 2015). Most of the clinical studies of VNS defaulted to the presence of concurrent drug therapy (Labar, 2004; García-Navarrete et al., 2013), and some of them controlled drugs as study variables, confirming the efficacy of VNS- AEDs combination (Révész et al., 2018). Transcranial electrical stimulation (tES) techniques, by direct or alternating current (tDCS/tACS), are promising neuromodulation tools for non-invasive treatment of epilepsy (Simula et al., 2022). Several studies revealed the potential of combining tES with AEDs, demonstrating that the addition of lorazepam or diazepam to tDCS stimulation can be effective in increasing the antiepileptic effect of the stimulation. Therefore, combined physical modulation with medication may reduce the dose of drugs and thus the side effects of the medication (Dhamne et al., 2015; Regner et al., 2020).

Gastrodin (GTD) the main bioactive constituent of *Gastrodia elata*, can exert antiepileptic effects in animal models by attenuating oxidative stress damage and reversing epilepsy-induced hyperactivation of cell membrane ion channels (Shao et al., 2017; Liu et al., 2018; Yavarpour-Bali et al., 2019). Nonetheless, due to the very inefficient spontaneous entry of GTD into the CNS, it has limited efficacy in controlling epilepsy when used alone in the clinic and may need to be combined with other therapies to be more effective (Wang et al., 2022). Thus, we propose a novel treatment for epilepsy using FUS combined with gastrodin. Firstly, kainic acid (KA) was used to induce seizures in mouse to investigate the antiepileptic effect of FUS combined with GTD. The relationship between GTD concentration in hippocampal tissue and seizure severity was evaluated through high-performance liquid chromatography (HPLC) assay. Then, the underlying mechanism of FUS combined with GTD was studied using Evans blue (EB) dye and 9.4T magnetic resonance imaging (MRI). Finally, we assessed the safety of combined therapy using H&E staining and 9.4T MRI. The results demonstrated that FUS combined with GTD is a promising treatment modality for epilepsy, providing a new therapy method for CNS disorders.

## Materials and methods

### Animals

C57BL/6J mice (adult male, weighted 18–22 g, Vital River Laboratory Animal Technology Co., Ltd., Beijing, China) are kept under controlled 24 ± 1°C temperature, 55 ± 5% humidity, and a 12-h illumination cycle, with free access to food and water. All animal experimental protocols (certificate number: SIAT-IRB-150213-YGS-ZHR-A0094-2) were approved by the IACUC of Shenzhen Institute of Advanced Technology, Chinese Academy of Sciences. We strictly adhered to the guidelines for animal experimentation and minimized animal suffering throughout the entire study.

### Electroencephalogram (EEG) electrode installation

Mice were fixed on a stereotaxic apparatus after isoflurane anesthesia, and EEG electrodes were thoroughly screw-winded on the skull localized to the hippocampus (−1.8 mm from Bregma; 1.8 mm left hemisphere); then the ultrasound collimator was affixed together with electrodes using dental ray resin. The recording EEG electrode was implanted on the left parietal bone (−1.8 mm from Bregma; 3.0 mm left hemisphere). While the earth wire was on the right occipital and the reference on the frontal skull. The mice were managed to recover for at least 72 h after electrode installation before performing follow-up studies.

### Establishment of an epilepsy model

EEG electrodes on the heads of the mice were connected to an EEG recorder. A 10-min EEG was recorded as baseline. A KA intraperitoneal (i.p.) (1 mg/mL in saline, 15 mg/kg injected, Sigma-Aldrich, USA) model was established. Generalized seizures, defined as the simultaneous occurrence of high-amplitude, high-frequency discharges in all channels, occurred 10 to 20 min after the KA injection. Generalized seizure counts and duration were assessed evaluation.

### Focused ultrasound procedure

**FIGURE 1:**
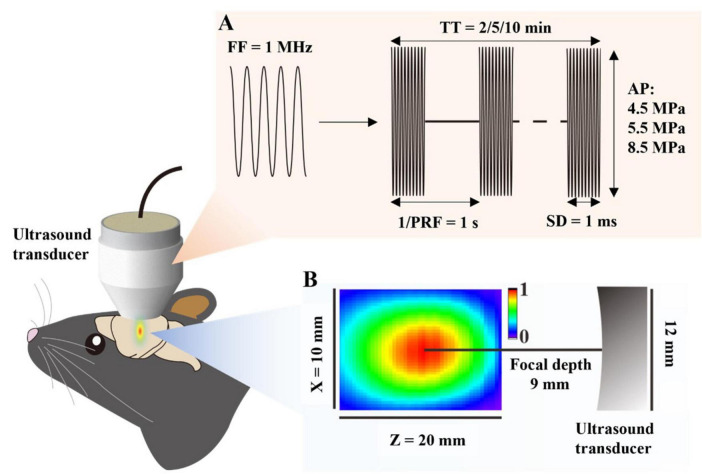
Schematic of combined therapy with FUS and GTD. **(A)** Schematic diagram of ultrasound parameters. **(B)** The distribution of the acoustic field. The ultrasound transducer has a diameter of 12 mm and a focal depth of 9 mm. FF, fundamental frequency, PRF, pulse repetition frequency, SD, sonication duration, AP, acoustic pressure, TT, total time.

A 1 MHz ultrasound transducer was used in this study (Guangzhou Doppler Electronic Technologies Co., Ltd.). Mice were induced anesthetized by 3% isoflurane for 5 min then maintained by 1.5% isoflurane of 1.0 L/min mixed gas on heating pad. Their skulls were exposed and positioned to the left hippocampus. A 3D-printed collimator designed based on the acoustic properties of the transducer was subsequently centered over the left hippocampus and affixed to the skull using a dental tray resin. The coupling agent was filled in the space inside the collimator. A wearable ultrasound transducer was then mounted on the collimator. All procedures were performed under sterile conditions (Figure 1).

An ultrasound signal generator (RIGOL, China) was used to drive the preset ultrasound parameters (Table 1). After the ultrasound insonation was completed, the mice were removed from anesthesia mask and were observed for behavioral abnormalities. Finally, the whole brain specimens of the mice were collected for permeability assay, or for safety assessment as described below.

**TABLE 1:** Acoustic parameters of FUS-GTD combination treatment.

No.	FF (MHz)	PRF (Hz)	DC (%)	Insonation time (min)	Acoustic pressure (MPa)	I_SPTA_ (mW/cm^2^)
1	1	1	0.1	5	4.50	164.3
2	1	1	0.1	5	5.50	245.5
3	1	1	0.1	5	8.50	586.4
4	1	1	0.1	2	5.50	245.5
5	1	1	0.1	10	5.50	245.5

FF, fundamental frequency; PRF, pulse repetition frequency; DC, duty cycle; I_SPTA_, spatial-peak temporal average intensity.

### FUS combined with GTD for seizure control

The KA-induced epilepsy mice were divided into five groups in this study: sham, FUS, saline + sham, GTD + sham and GTD + FUS. The saline group received saline instead of GTD for treatment, the GTD + sham group received GTD and sham FUS insonation, and the GTD + FUS group received GTD and FUS insonation.

GTD (10 mg/mL, 100 mg/kg; Aladdin) or saline (10 mL/kg, in the saline group) was injected i.p., and a 10-min FUS (sham) insonation was performed under anesthesia (ultrasound parameters: fundamental frequency = 1 MHz; PRF = 1 Hz; DC = 0.1%; total time = 10 min; acoustic pressure = 5.50 MPa) 10 min after injection.

### Electroencephalogram recording and analysis

Electroencephalogram signals were recorded by an EEG recording system (SOLAR 1848, Solar Electronic Technologies Co., Ltd, China. Sampling rate 500 Hz). The bandpass filter for data acquisition was set between 0.01 Hz and 70 Hz. We assessed the antiepileptic effect of FUS-GTD combination therapy by analyzing the number of epileptic EEG occurrences and the duration of epileptic EEG. We further employed the Pwelch function in MATLAB to evaluate the power spectral density (PSD) of the data to describe the effects in different oscillation frequency bands (δ oscillation of below 4, θ of 4–8, α of 8–12, β of 12–30 Hz, and γ oscillation of over 30 Hz).

#### Detection of GTD concentration using high-performance liquid chromatography (HPLC)

Mice were deep anesthetized to collect brain and plasma samples for HPLC. Blood samples were obtained via cardiac puncture and stored in heparinized sodium tubes at room temperature. Plasma samples were separated by centrifugation. The brain was dissected into hemispheres, weighed, labeled, and stored at −80°C. Homogenized brain and plasma samples were deproteinized in 80% methanol and centrifuged. Analyses were conducted after transferring the supernatant to a new tube, drying it under nitrogen, and reconstituted it with 20% methanol (Jiang et al., 2013; Zhao et al., 2019). Blank samples were similarly prepared for calibration curves, with brain and plasma calibration concentrations set between 0 and 50 μg/mL and 0 and 10 μg/mL, respectively, organized from the lowest to highest concentration for analysis.

The analysis utilized a K2025 HPLC system (Wooking Instruments, Shanghai, China) with a mobile phase of 95% ACN (A) and 1% methanol containing 0.05% phosphoric acid (B). It consisted of five gradient programs: 0 to 1 min (98%) B, 1 to 11 min (92% B), 11 to 17 min (70% B), 17 to 22 min (10%) B, and 22 to 30 min (98% B). Flow rates of 1 mL/min were set, and detection was performed at a wavelength of 221 nm with a column temperature of 25°C. The injection volume for each sample was 15 μL. Chromatographic data analysis was conducted using the built-in module of the Wooking software, with manually adjusted peak areas.

#### Brain sample collection

The mice were deep anesthetized by 5% isoflurane mixed gas on the dissecting board. The thoracic cavity was cut open using scissors. After the heart was dissected from the right atrium, 20 mL of saline was injected continuously into the left ventricle, followed by 20 mL of paraformaldehyde (PFA) at 4%. The brain collected was preserved in 4% PFA.

#### Permeability assessment

Mice were induced anesthetized by 3% isoflurane for 5 min then maintained by 1.5% isoflurane of 1.0 L/min mixed gas on heating pad to help visualize the tail veins. An injection of Evans blue (EB, 4%, 0.2 mL, Sigma-Aldrich, USA) was given to the mice through their tail veins, followed by stereotactic manipulation of the mouse brain.

Brain sections were used to observe the distribution of EB. After a 24-h fixation in 4% PFA, the brain tissue was removed from the test tube and the residual PFA solution was rinsed. The brains were then placed in a brain-sectioning mold, sliced into 1 mm-thick slices, then transferred onto slides. The brain slices were sealed with light-curing resin and coverslips. Images of sections were captured using a camera.

#### H&E staining

4% PFA-fixed brain samples were embedded in paraffin, cut into 5 μm thick slices and mounted on poly-L-lysine-coated glass slides. Sections were deparaffinized and hematoxylin-stained. Nuclei were differentiated using 1% hydrochloric acid-alcohol solution for 20 s and nuclear staining was enhanced by immersion in 1% ammonia for 30 s. Each specimen was stained using 1 drop of 1% eosin for 5 min, washed with distilled water and dehydrated using a gradient of ethanol. The tissue was made transparent by immersion in dimethyl benzene and finally the slides were sealed with neutral gum. The prepared slides were photographed using a slide scanner (NanoZoomer S60, Hamamatsu, Japan).

#### MRI

A 9.4T MRI system (uMR 9.4T; United Imaging Life Science Instrument, China) was used to confirm FUS-mediated drug permeability and assess the safety of FUS.

To confirm drug permeability, mice were injected with a 0.2 mL mixture of gadolinium-DTPA (Gad, 938 g/mol, Magnevist) and 4% EB via the tail vein, as described above. The mouse head was further scanned using a T1-weighted fast rotational echo (FSE) sequence before and after the FUS treatment, and the mice were anesthetized with isoflurane throughout the scan. At the end of the scan, the mice were sacrificed, and their brains were collected after perfusion and sectioned to observe the distribution of EB.

The T1-weighted FSE sequence was set as follows: repetition time (TR) = 500 ms, echo time (TE) = 5.14 ms, echo train length (ETL) = 4, bandwidth (BW) = 300 Hz/pixel, field of view: 17 mm^2^ × 14 mm^2^, matrix size 352 × 288, slice thick = 500 μm, and gap = 100 μm.

To evaluate the safety of FUS treatment, we divided the mice into the FUS and Sham groups. The mice were scanned at three different time points (before FUS, after FUS, and 72 h after FUS insonation) with T1-weighted FSE and T2-weighted FSE sequences from 9.4T MRI to assess the safety of FUS.

ebETL = 3, BW = 220 Hz/pixel, field of view: 20 mm^2^ × 20 mm^2^, matrix size 208 × 208, slice thick = 500 μm, and gap = 100 μm.

The T2-weighted FSE sequence was set as follows: TR = 3,000 ms, TE = 46.34 ms, ETL = 13, BW = 220 Hz/pixel, field of view: 20 mm^2^ × 20 mm^2^, matrix size 208 × 208, slice thick = 500 μm, and gap = 100 μm.

#### Intracranial temperature measurement

A metal probe from a TES 1310 type-k thermometer was securely fixed on a manipulator arm and implanted into the cranium of mouse, which was fixed in place using a stereotactic head frame at a 45° angle through pre-drilled holes to ensure the temperature probe was located near the insonation region. After waiting for the thermometer’s digits to stabilize substantially, temperature digits were recorded at 1-min intervals over a 10-min period as a baseline. Subsequently temperature was recorded during the 10-min ultrasound treatment. Recording continued for an additional 10 min after ultrasound was switched off.

### Statistical analysis

Prism software 9.5 (GraphPad, USA) was selected to perform statistical analyses in this paper. For two-group comparisons, Student’s *t*-test was used. For comparisons between three groups, one-way ANOVA followed by Tukey’s *post-hoc* test was used to determine statistical significance. Values reported in the text are expressed as the mean ± standard error of the mean.

## Results

### FUS combined with GTD for seizures inhibition

**FIGURE 2:**
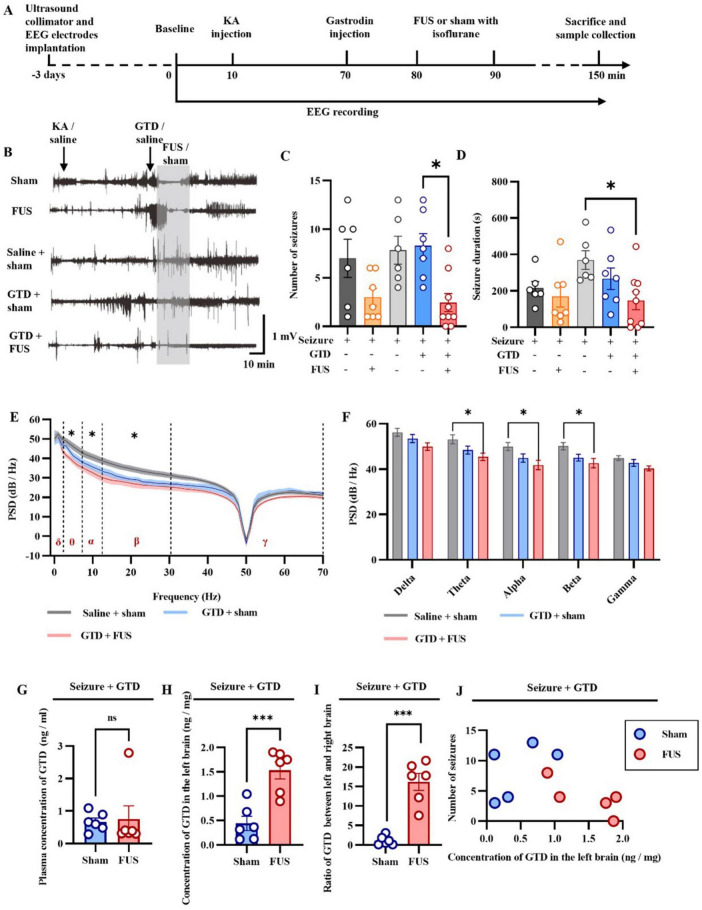
FUS combined with GTD for seizure control. **(A)** Flow chart of the study. **(B)** Representative diagram of an exemplar epileptic electroencephalogram. **(C)** Seizure counts (sham, *n* = 6; FUS, *n* = 7; saline + sham, *n* = 6; GTD + Sham, *n* = 7; GTD + FUS, *n* = 7). **(D)** Statistical graph of seizure duration (sham, *n* = 6; FUS, *n* = 7; saline + sham, *n* = 6; GTD + Sham, *n* = 7; GTD + FUS, *n* = 7), one-way ANOVA. **(E)** Power spectral density values at different frequency bands. **(F)** Statistical graph of PSD values at different frequency bands (saline + sham, *n* = 6; GTD + Sham, *n* = 5; GTD + FUS, *n* = 5). **(G)** GTD concentration in the blood. **(H)** GTD concentration in the left brain. **(I)** The ratio of GTD concentration between the left and right brain. **(J)** Correlation between GTD concentration in the left brain and the number of seizures. *p < 0.05, ***p < 0.001, ns = non-significant difference.

We used the rodent kainic acid-induced epilepsy model and assessed seizure severity by EEG to investigate the antiepileptic effect of FUS combined with GTD (Figure 2A). In this study, KA intraperitoneally injection-induced seizure mice were randomly categorized into five groups: sham, FUS, saline + sham, GTD + sham and GTD + FUS. GTD was injected i.p. before FUS. A 10-min and 5.5-MPa-acoustic-pressure FUS was targeted on the left hippocampus to exert antiepileptic effects in combination with GTD therapy. The results showed that combined FUS + GTD treatment reduced the seizure counts and duration by more than 50% in comparison to the saline group (Figures 2B–D). Seizure counts of GTD + FUS group were significantly less than counts of GTD + sham group. There was also a trend toward shorter seizure durations. Neither the GTD + Sham nor the Saline groups had significantly different seizure numbers or durations (Figures 2B–D). These results show that the combination of FUS + GTD reduced the seizure counts and duration. We further evaluated the antiepileptic effect of FUS combined with GTD by analyzing the power spectral density (PSD). The results showed that the PSD values in several frequency bands were reduced in the GTD + sham and GTD + FUS groups in comparation to the saline group. The reductions in the PSD values of the θ, α, and β frequency bands reached significant levels between GTD + FUS and saline groups (Figures 2E, F).

We hypothesized that FUS acting in the hippocampus enhances the antiepileptic effect by increasing GTD concentration. We examined the GTD concentration in brain tissue and found that the GTD concentration in the left side (FUS-treated side) of the brain in the GTD + FUS group was significantly higher than that in the GTD + Sham group (Figure 2H). The comparison of GTD concentrations between the left and right brains suggested that the GTD of left brain in the GTD + FUS group was about 15 times more than right side. In contrast, in the GTD + sham group, the amount of GTD in the left and right brains was essentially the same (Figure 2I). And between each group, GTD concentrations in the circulatory system were consistent (Figure 2G). We further investigated the relationship between GTD concentration in the left brain and the number of seizures (Figure 2J). In the GTD + FUS group, mice with higher GTD concentration in the FUS treated side of the brain had fewer seizures, and all animals in this group were distributed in the lower right corner of the graph, implying a negative correlation between GTD concentration and seizure counts. These results suggest that FUS can increase the concentration of GTD in the brain and thus exert antiepileptic effects.

### Optimized FUS parameters for drug permeability

**FIGURE 3:**
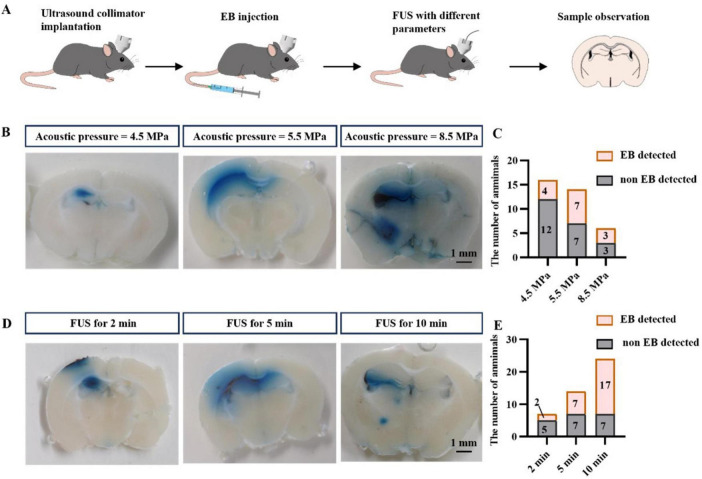
FUS promotes drug entry into brain tissue. **(A)** Flow chart of the study. **(B)** Representative graphs of EB entry into brain tissue at different acoustic pressures. **(C)** Statistical graphs of EB entry into brain tissue at different acoustic pressures. **(D)** Representative graphs of EB entry into brain tissue at different insonation times. **(E)** Statistical graphs of EB entry into brain tissue at different insonation times.

As shown in Figure 3, to investigate the appropriate parameters for FUS to promote drug permeability, we first injected EBs into mice through the tail vein to mimic large molecules of water-soluble drugs in plasma. Subsequently, we performed ultrasound intervention on the brains of mice using different parameters (including sound pressure and ultrasound intervention time). The effects of various ultrasound parameters on drug permeability were assessed by evaluating the EB content in brain tissues. These results provide a parametric basis for the rest of our experiments.

We stimulated the left hippocampus of mice with FUS at different acoustic pressures (4.5, 5.5, and 8.5 MPa, Figure 3B), collected brain tissue and assessed the number of samples in which EB was found. We found that EB was detected in only 25% of the samples treated with FUS at 4.5 MPa (4/16), whereas FUS at 5.5 MPa increased the percentage of EB detection to 50% (7/14). Although FUS up to 8.5 MPa also resulted in a 50% EB detection rate (3/6), we chose 5.5 MPa FUS in our follow-up study for the safe use of ultrasound.

We further investigated the effect of insonation time on drug permeation efficiency using FUS. Three different insonation times were used: 2 min, 5 min, and 10 min, and the results are shown in (Figures 3D, E). As previously observed, the EB detection rate was 50% (7/14) after 5 min FUS, only 28% (2/7) with 2 min FUS, and increased to 70% (17/24) with 10 min FUS. In conclusion, these results suggest that the efficiency of FUS in facilitating drug entry into brain tissue is correlated with the sound pressure of FUS and the duration of the intervention. We combined the optimal parameters (5.5 MPa, 10 min insonation time) for the remaining studies.

### 9.4T MRI observed FUS-enhanced drug permeability

**FIGURE 4:**
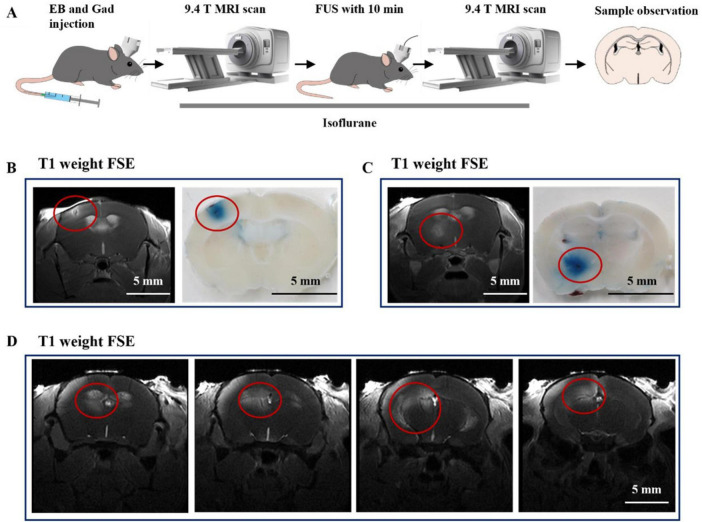
The underlaying mechanism of combined therapy. **(A)** Flow chart of the study. **(B)** Representative images of FUS-induced Gad-DTPA distribution in the cerebral cortex (*n* = 6). **(C)** Representative images of FUS-induced Gad-DTPA distribution in the deep brain (*n* = 6). **(D)** Representative images of FUS-induced Gad-DTPA distribution in the hippocampus (*n* = 6).

9.4T MRI provides high-resolution and fine images for brain imaging in mice. It is necessary to use MRI to further investigate the spatial-temporal properties of FUS-promoting drug permeability. Gadolinium DTPA (Gad-DTPA), a commonly used contrast agent in MRI, has a relative molecular weight of 938.02 and is unable to enter the brain parenchyma under physiological conditions. Therefore, we used 9.4T MRI to visualize the distribution of Gad-DTPA in the brain after FUS intervention after injecting Gad-DTPA intravenous. In addition, we added 4% EB to the Gad-DTPA solution to check the consistency of the gadolinium contrast agent on MRI imaging and the EB-stained areas in the slices (Figure 4A). We selected a cortex area (Figure 4B) and a deep brain region (Figure 4C) for observation. As shown in (Figures 4B, C), the signals in the FUS-stimulated region were higher than those in other regions, indicating that Gad-DTPA could rapidly enter the brain tissue after 10 min of FUS treatment.

Subsequently, we targeted FUS to the hippocampus to observe the drug-passage-promoting effects. As shown in Figure 4D, we found that FUS could mediate the distribution of Gad-DTPA in different parts of the hippocampus. These results reveal the feasibility of enhancing the efficacy of treating neurological disorders by combining drugs with FUS.

### Safety of FUS treatment

**FIGURE 5:**
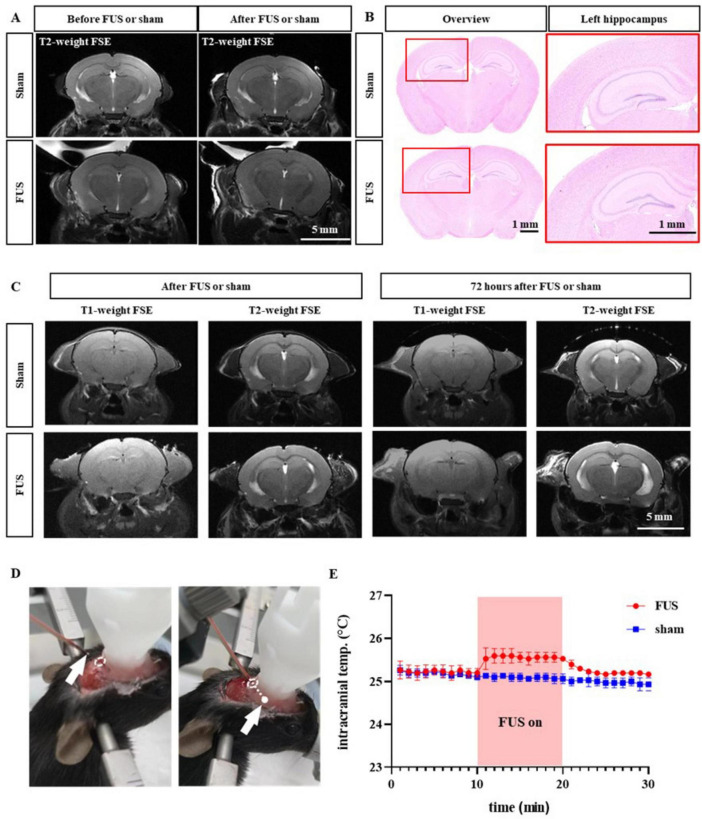
Safety assessment of combined therapy. **(A)** Representative magnetic resonance imaging (MRI) before and after sham or FUS treatment. **(B)** Representative H&E staining images in the sham and FUS groups. **(C)** Example MRI pictures 72 h after sham or FUS treatment. **(D)** Type-K thermocouple probe was implanted into intracranial insonation region. **(E)** Intracranial temperature changes before, during and after ultrasound treatment.

As shown in Figure 5, we further evaluated the safety of FUS-enhanced drug permeability using 9.4T MRI and H&E staining. We performed 9.4T MRI imaging before and after FUS insonation, which showed no tissue damage such as hemorrhage or necrosis in the hippocampus, the FUS target area, or any other areas. We then sacrificed and collected the brains of the mice and applied H&E staining to assess the safety of FUS at the tissue level. The cells inside and outside the insonated area did not show any pathological changes, such as cellular edema or necrosis. Finally, we performed the MRI after 72 h of insonation with FUS, which showed no FUS-induced damage such as hemorrhage or edema. In order to more accurately reflect the temperature rise induced by ultrasound, we implanted a temperature probe into the intracranial insonation region of mice (Figure 5D). We found that 10-min ultrasound treatment at an ambient temperature of 21.2°C resulted in an approximately 0.5°C increase in the temperature of intracranial insonation region. Importantly, the temperature rapidly returned to the baseline level after the ultrasound treatment was stopped (Figure 5E). Therefore, we concluded that the use of FUS to enhance drug permeability is a safe means of combining neuromodulation and drug therapy.

## Discussion

In this study, we propose a new therapy method to enhance antiepileptic efficacy using FUS combined with systemic GTD injection. The results showed that FUS combined with GTD treatment was effective in reducing seizures (Figure 2). The underlying mechanism may be that FUS promotes the entry of GTD into the brain parenchyma by specific parameters. HE staining and 9.4T MRI verified the safety of the combined treatment at different time points. Therefore, this research provides a novel therapy method for neurological disorders.

The effect of inhibition of abnormal discharges produced by focused ultrasound when applied to resected focal tissues in epileptic patients has been demonstrated in our earlier study and explained in terms of excitatory/inhibitory output ratio modulation (Lin et al., 2020). However, for clinical epileptic patients, basic antiepileptic drugs are mandatory, regardless of whether surgical or neuromodulatory treatment is implemented (Thijs et al., 2019). Hence, it will be of vitally practical to focus on the effect of FUS treatment combined with drugs and to search for a therapeutic protocol that can enhance the efficacy of the combination. In our study, FUS stimulation of hippocampal epileptic foci alone already produced seizure suppression (Figures 2C, D), and the antiepileptic effect reached a significant level after combining GTD drugs. The improved efficacy was highly synchronous with the elevated brain tissue GTD concentration revealed by HPLC (Figures 2G–J), suggesting that ultrasound may have contributed to the entry of GTD into the brain tissue, which in turn exerted a combined therapeutic effect. Noticeably, the application of GTD alone does not inhibit seizures *in vivo* as GTD does not spontaneously enter the brain tissue (Figures 2C, D).

When FUS is applied to the nervous system, the specific effects produced are closely related to the acoustic parameters. The application of different stimulation parameters can specifically modulate depression, epilepsy, dementia or traumatic brain injury (Sarica et al., 2022). In our study, we found that the effect of FUS in promoting the entry of drugs in the circulation into brain tissue and thus enhancing the suppression of epilepsy was closely related to ultrasound parameters such as acoustic pressure and insonation time (Figure 3). Increasing the acoustic pressure within a certain range significantly increased the detection rate of EB dye in the brain parenchyma. In addition, we discovered that the insonation time of FUS contributes significantly to drug entry into the brain parenchyma. Although EB dye could be observed in the brain parenchyma after 2 min of FUS in approximately 30% of individuals, we believe that this effect is not sufficient to support a stable enhancement effect. In comparison, 10 min of FUS insonation increased the detection of EB dye to more than 70%. Considering that prolonging the duration of action may pose additional safety risks, we used the 10-min insonation time FUS parameter for the remainder of the study. In the *in vivo* experiments we conducted, FUS combined with GTD when using the above parameter combinations elevated the GTD concentration in the intervention area to 15-fold of that on the contralateral side and significantly suppressed seizures induced by intraperitoneal injection of KA. The fundamental frequency and duty cycle are two constant parameters in this study. The fundamental frequency (FF) is an inherent characteristic of ultrasonic transducers. The range of the fundamental frequency (can up to serval MHz) far exceeds the neuron’s natural rhythm (few Hz to a few hundred Hz) (Ivanovski et al., 2024). Since neurons cannot differentiate between various FFs within this wide range, the FF parameter is not investigated in this study. As for duty cycle (DC), although a higher value can potentially enhance efficacy by delivering more energy to the biological tissue (Hölscher et al., 2013; Kubanek et al., 2018), it also increases the risk of rapid heat accumulation in the insonation area. Therefore, the DC parameter has been set to a relatively low level (0.1%) to ensure safety in our study. In future research, we will further explore the role of different ultrasonic parameters in combination therapy.

Our results suggest that ultrasound can facilitate GTD entry into brain tissue to exert combined therapeutic effects. Regarding possible mechanisms, ultrasound could alter membrane permeability by modulating mechanosensitive channels or inducing the secretion of vasoactive agents. Several mechanosensitive ion channels exist in vascular endothelial cells, such as Piezo1 and TRPA1 (Fels and Kusche-Vihrog, 2020; Lai et al., 2021). These ion channels are sensitive to variations in mechanical forces in surroundings, which can be modulated by mechanical forces such as ultrasound and shockwaves to decrease the expression of occludin, claudin-5, and ZO-1 in vascular endothelial cells (ECs), leading to downregulation of the tight junction (TJ) complex between ECs (Sheikov et al., 2008; Ranade et al., 2014; Liao et al., 2020; Newman et al., 2024). Furthermore, it was found that ultrasound also hyperpolarises the cell membrane by increasing shear stress-dependent potassium channels in vascular endothelial cells. The production of nitric oxide (NO) occurs when nitric oxide synthase is activated in vascular endothelial cells. Nitric oxide (NO) causes vasodilatation and greatly increases vessel permeability (Hoger et al., 2002; Altland et al., 2004; Krizanac-Bengez et al., 2004; VanBavel, 2007). In addition, ultrasound reduces the stability of the ECs tight junction by inducing an increase in protein kinase B (Akt) phosphorylation and activating the PI3K/Akt pathway (Jalali et al., 2010). We suggest that FUS at the specific parameters in this study likely activated mechanosensitive ion channels on the ECs, triggering an increase in NO, down-regulation of occludin, claudin-5, and ZO-1, and a decrease in local ECs tight junction stability as a mechanism for the subsequent increase in drug permeability. However, the detailed mechanisms involved in this study still need to be investigated. Our subsequent focus is on the responsiveness of cell membrane ion channels to ultrasound under specific parameters (Ye et al., 2018) and the molecular pathways mechanisms of tight junction disturbance.

In this study, we evaluated the safety of FUS combined with drug therapy using 9.4T magnetic resonance imaging, described the spatial and temporal properties of FUS-mediated drug permeability, and checked MRI imaging against pathological sections for concordance (Figure 5). Our study confirms that a balance of enhanced efficacy and guaranteed safety of ultrasound combined with drugs can be achieved under specific parameters. Clinical attempts to utilize transcranial ultrasound stimulation for the treatment of epilepsy have been reported (Bubrick et al., 2024) confirming its safety and effectiveness. Typically, ultrasound is paired with microbubbles for drug delivery, especially when targeting brain tumors, and this method is progressively being adopted in human trials (Meng et al., 2021). Our study reveals the combination of the two approaches, hinting at potential applications in treating epilepsy or brain disorders like Parkinson’s disease. However, it’s crucial to know that acoustic characteristics of human skull differ significantly from those of mice. Thus, the balanced parameters for human trials need further exploration for clinical applications.

We explored the efficacy and relationship between local GTD concentration and the efficacy of FUS in treating epilepsy; however, this study has some limitations. The mechanism of FUS combined drug action at the cellular level needs to be further investigated. Second, although we evaluated the effect of ultrasound-enhanced stimulation of local drug concentration using several acoustic parameters, the composition of ultrasound parameters is complex and needs to be continuously optimized in further studies. Furthermore, the experimental design of the present study focused on the acute phase outcome. Monitoring the increase in intracranial temperature and the observing the injuries by MRI and H&E staining have verified that a single application of ultrasound did not lead to acute injury. Regarding the neural activity caused by ultrasound, we did not observe any behavioral abnormalities in healthy mice that underwent ultrasound intervention. Certainly, the duration of our experimental program was short, and further investigation is required to understand the effects of repeated ultrasound interventions over an extended period of time.

## Data Availability

The original contributions presented in this study are included in this article/supplementary material, further inquiries can be directed to the corresponding authors.
